# Conserved and novel functions of programmed cellular senescence during vertebrate development

**DOI:** 10.1242/dev.138222

**Published:** 2017-01-01

**Authors:** Hongorzul Davaapil, Jeremy P. Brockes, Maximina H. Yun

**Affiliations:** 1 Institute of Structural and Molecular Biology, Division of Biosciences, University College London, London WC1E 6BT, UK; 2 Institute of Ophthalmology, University College London, London EC1V 9EL, UK

**Keywords:** Cellular senescence, Axolotl, *Xenopus*, TGFβ, Cement gland, Kidney

## Abstract

Cellular senescence, a form of stable cell cycle arrest that is traditionally associated with tumour suppression, has been recently found to occur during mammalian development. Here, we show that cell senescence is an intrinsic part of the developmental programme in amphibians. Programmed senescence occurs in specific structures during defined time windows during amphibian development. It contributes to the physiological degeneration of the amphibian pronephros and to the development of the cement gland and oral cavity. In both contexts, senescence depends on TGFβ but is independent of ERK/MAPK activation. Furthermore, elimination of senescent cells through temporary TGFβ inhibition leads to developmental defects. Our findings uncover conserved and new roles of senescence in vertebrate organogenesis and support the view that cellular senescence may have arisen in evolution as a developmental mechanism.

## Abstract (precis)

**Summary:** Cellular senescence is an intrinsic part of the developmental programme in amphibians and has a conserved role in vertebrate organogenesis.

## INTRODUCTION

Cellular senescence is state of permanent cell cycle arrest that is induced in response to various stresses, including telomere erosion, DNA damage and oncogene activity (Serrano et al., 1997; Bodnar et al., 1998; Campisi and d'Adda di Fagagna, 2007). As such, it constitutes a powerful anti-tumourigenic mechanism (Braig et al., 2005; Collado et al., 2005). In addition to a stable cell cycle arrest, senescent cells exhibit high levels of senescence-associated β-galactosidase (SAβgal) activity, a well-established senescent cell marker (Dimri et al., 1995); increased cell size and flattened morphology; expansion of mitochondrial and lysosomal networks; chromatin and nuclear rearrangements; and increased expression of hallmark regulatory proteins, such as p53, p21 and p16 (Campisi and d'Adda di Fagagna, 2007; Rai and Adams, 2013; Burton and Krizhanovsky, 2014). Furthermore, senescent cells acquire a senescence-associated secretory phenotype (SASP), which comprises growth factors, cytokines, chemokines and matrix remodelling proteins, and can lead to inflammation, alterations in tissue microenvironment and paracrine transmission of cell senescence (Campisi and d'Adda di Fagagna, 2007; Kuilman and Peeper, 2009; Nelson et al., 2012; Acosta et al., 2013).

Despite its role in tumour suppression, cellular senescence can have negative effects on biological processes. This is of particular relevance to ageing, as senescent cells accumulate in various tissues as organisms age (Yun, 2015), thereby contributing to a number of age-related pathologies (Baker et al., 2011; Baker et al., 2016; van Deursen, 2014), and promoting loss of regenerative capacity in the muscular and haematopoietic systems, among others (Sousa-Victor et al., 2014; Chang et al., 2016). Beyond ageing, senescent cells have been found to promote tumourigenesis in specific contexts (Krtolica et al., 2001; Laberge et al., 2012). These observations have recently revived discussions on the evolutionary rationale for cellular senescence and it has been suggested that senescent cells could have positive physiological roles. Indeed, transient induction of senescence has been shown to contribute to restriction of fibrosis in skin wounds (Jun and Lau, 2010), liver (Krizhanovsky et al., 2008) and heart (Zhu et al., 2013), as well as to facilitating wound closure (Demaria et al., 2014). Importantly, cellular senescence has been recently described in non-pathological states, including development (Chuprin et al., 2013; Muñoz-Espín et al., 2013; Storer et al., 2013) and regeneration (Yun et al., 2015). During mammalian development, senescent cells are found throughout the embryo during restricted time windows, in structures such as the embryonic kidney, the endolymphatic sac of the inner ear, the neural roof plate and the apical ectodermal ridge of the limb (Muñoz-Espín et al., 2013; Storer et al., 2013). Developmental senescence is transient, and its programmed elimination involves immune-mediated clearance (Muñoz-Espín et al., 2013; Storer et al., 2013). Mechanistically, it is dependent on p21, the genetic disruption of which leads to loss of senescence accompanied by developmental abnormalities in various structures. This suggests that senescent cells might play a role in instructing growth, patterning and tissue remodelling during development (Muñoz-Espín et al., 2013; Storer et al., 2013), although more evidence is needed. These findings have led to the proposal that developmental senescence may have pre-dated the evolutionary origin of stress-induced senescence (Muñoz-Espín et al., 2013; Storer et al., 2013). However, the degree of conservation of developmental senescence through phylogeny, as well as the extent of its functions during development, remain unknown.

Here, we demonstrate that cellular senescence is an intrinsic part of amphibian development. Furthermore, we identify conserved and novel features of developmental senescence during degeneration and remodelling of crucial structures, such as the pronephros, the cement gland and oral cavity. Our findings extend the participation of cellular senescence in developmental processes from amniotes to amphibians, and suggest it could be a feature of all vertebrates, supporting the view of an early origin for developmental senescence during evolution.

## RESULTS

### Cellular senescence in axolotl development

**Fig. 1.**
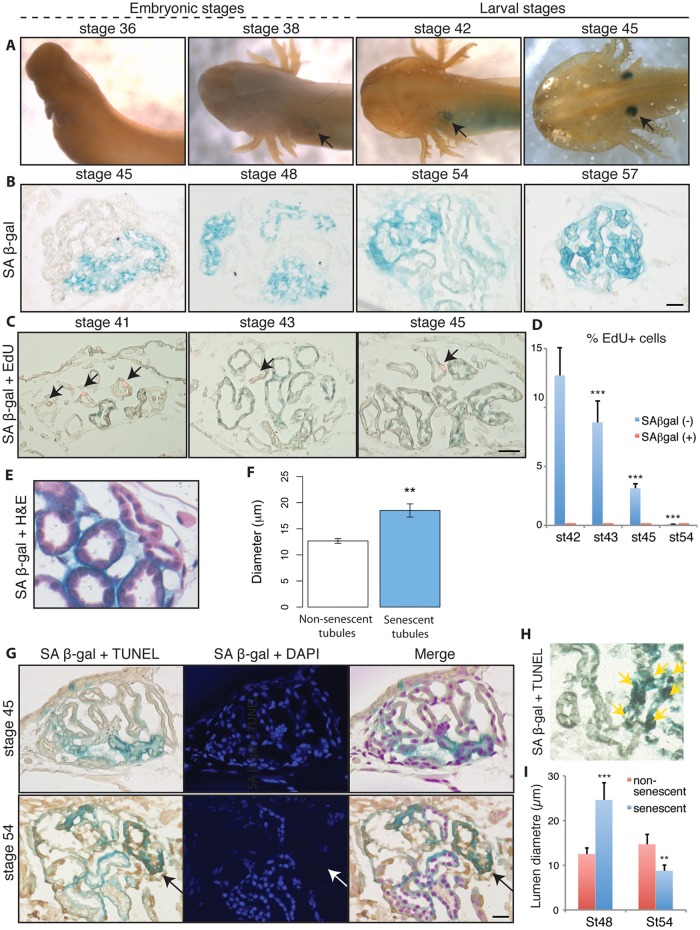
**Programmed cellular senescence during the development of the axolotl pronephros.** (A) Whole-mount senescence-associated β-galactosidase (SAβgal) staining of axolotl embryos at the indicated stages (*n*=8 per stage). Arrows indicate SAβgal staining in the developing pronephros from stage 38 onwards. (B) Representative cryosections of whole embryos following SAβgal staining at the indicated stages. The senescence spreads with concurrent degeneration of the pronephros at stage 57 (*n*=8 per stage). Scale bar: 50 µm. (C) Representative image of SAβgal/EdU co-staining of whole embryos, 48 h after EdU administration. EdU^+^ cells are indicated (arrows). No SAβgal^+^/EdU^+^ cells were found in any of the samples analysed (*n*=5). Scale bar: 50 µm. (D) Percentage of EdU-positive cells within SAβgal-positive or -negative cell populations in pronephric tubules at the indicated stages (*n*=4, ***P<0.001). (E) Representative paraffin wax-embedded section of axolotl pronephros following SAβgal/Haematoxylin and Eosin co-staining. Cells have enlarged and flattened morphology within SAβgal^+^ tubules. (F) Quantification of average tubule diameter in non-senescent and senescent pronephric tubules (**P<0.01). (G) TUNEL assay in SAβgal whole-mount stained, paraffin wax-embedded sections. Senescent tubules undergo apoptosis (arrows) at late larval stages. TUNEL-positive pronephric tubules also have decreased nuclear staining (*n*=5). Scale bar: 50 µm. (H) Representative TUNEL staining in a SAβgal-stained cryosection of axolotl tissues at stage 54. TUNEL-positive nulei are absent (in black, yellow arrows) exclusively in senescent pronephric tubules. (I) Quantification of average lumen diameter in non-senescent and senescent pronephric tubules at the indicated stages (*n*=5, **P<0.01, ***P<0.001). Data are mean±s.e.m. in D,F,I.

To determine whether cellular senescence occurs *in vivo* during amphibian development, we performed senescence-associated β galactosidase (SAβgal) staining of whole axolotl (*Ambystoma mexicanum*) embryos followed by sectioning at various developmental stages. SAβgal had been validated for the detection of senescence in amphibians in earlier work, by analysis of marker expression in senescent cells in culture and *in vivo* (Yun et al., 2015). Notably, from stage 38 onwards, we identified an area of SAβgal-positive cells within a tubular structure that corresponded to the embryonic kidney: the pronephros (Fig. 1A,B). This was reminiscent of previous reports of programmed cellular senescence during development of the embryonic kidney in mammals: the mesonephros (Muñoz-Espín et al., 2013).

Vertebrates employ a succession of kidney forms throughout their lives for regulating water balance and waste disposal. These include the pronephros, mesonephros and metanephros (Carroll and Vize, 1996). In amniotes, the pronephros is a transient structure, whereas the mesonephros constitutes the principal embryonic kidney form. Following its degeneration, the mesonephros is succeeded by the metanephros, which remains functional through adulthood. By contrast, the pronephros is the main kidney form used throughout embryonic and larval stages in amphibians and fish, with the mesonephros constituting the functional adult kidney form (Carroll and Vize, 1996; Wingert and Davidson, 2008). Notably, despite varying in complexity, the amphibian pronephros and mammalian mesonephros share structural, developmental and molecular features, including the use of similar patterning and segmentation genes (Vize et al., 1997; Wingert and Davidson, 2008; Desgrange and Cereghini, 2015). Considering this, and in the light of our observations, we hypothesised that these structures could also share a similar mechanism of degeneration, based on the induction of cellular senescence.

Consistent with this idea, the SAβgal staining was first evident at stage 38, expanded within the structure during subsequent stages, and persisted until the complete degeneration of the pronephros at advanced stage 57 (Fig. 1, Fig. S1), the stage at which the hindlimb is fully developed (Nye et al., 2003). SAβgal staining in the pronephros is also described elsewhere (Villiard et al. 2016). At the cellular level, SAβgal-positive cells were always negative for proliferative markers (EdU incorporation, Fig. 1C,D) and exhibited other hallmarks of senescence, such as an enlarged morphology (Fig. 1E). This coincided with an increased tubule cell diameter (Fig. 1F) and with higher protein levels of the p53 tumour suppressor (Fig. S2), suggesting that these are indeed senescent cells.

The propagation of cellular senescence within the structure was directional, with tubules undergoing senescence following a caudal-to-cranial sequence (Fig. S1). Nonetheless, the duct, which is the caudalmost part of the pronephros, underwent senescence at later stages – from stage 50 onwards – as shown by co-staining with SAβgal and a pronephric duct-specific antibody (Brennan et al., 1998) (Fig. S3). Notably, the overall directionality of the senescence spreading coincides with the directionality of pronephros degeneration in amphibians (Fox, 1962), as well as with that of mesonephros degeneration in mammals (Davidson, 2008; Muñoz-Espín et al., 2013), which also progress in a caudal-to-apical manner. Indeed, senescent tubules eventually undergo a structural collapse, resulting in a significant decrease in lumen diameter (Fig. 1I), which is most evident from stage 54 onwards (Fig. 1, Fig. S1), the stage at which the axolotl pronephros is no longer functional and begins to degenerate (Haugan et al., 2010). This is in agreement with previous observations of marked decreases in pronephric tubule volume at the onset of degeneration (Haugan et al., 2010). The structural collapse of the pronephric tubules was accompanied by a marked recruitment of macrophages/monocytes (Fig. S4) and by a low recruitment of neutrophil/granulocytes, as assessed using α-naphthyl acetate esterase and naphthol AS-D chloroacetate esterase staining (Fig. S3). Importantly, we observed a significant induction of apoptosis within the senescent pronephric tubules at stage 54, as assessed by TUNEL staining in paraffin wax-embedded sections and cryosections (Fig. 1G,H), which is concomitant with macrophage infiltration (Fig. S4) and precedes the involution of the pronephros. It remains to be established whether macrophage recruitment precedes, or is a consequence of, the induction of apoptosis. Hence, our results raise the possibility that programmed cellular senescence promotes the degeneration of the axolotl pronephros through an apoptosis-dependent mechanism.

**Fig. 2.**
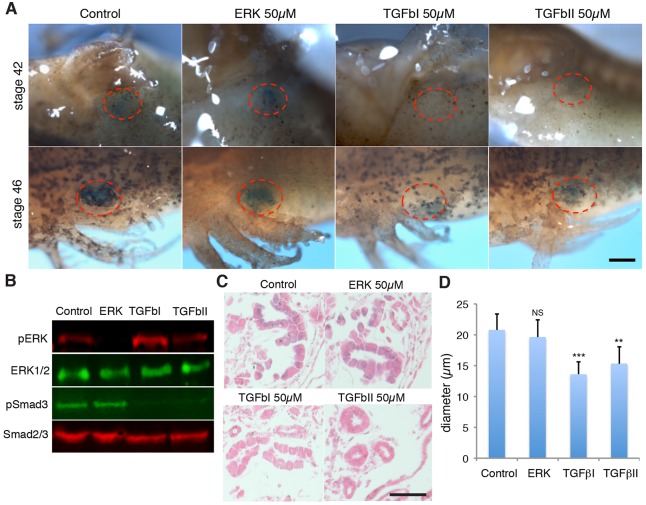
**Cellular senescence in the axolotl pronephros is independent of the ERK/MAPK pathway and dependent on TGFβ signalling.** (A) Whole-mount senescence-associated β-galactosidase (SAβgal) staining of axolotl embryos following treatment with the indicated inhibitors from stage 30 onwards. Red dotted lines indicate the pronephric area. Scale bar: 200 µm. (B) Western blot analysis of whole-embryo extracts following the indicated treatments. Phospho-ERK1/2 and phospho-Smad3 levels are decreased upon inhibition of ERK and TGFβ signalling, respectively, indicating that all inhibitors are functional (*n*=5). (C) Representative SAβgal/Eosin-stained paraffin wax-embedded sections of axolotl tissues from embryos exposed to the indicated treatments (*n*=6). Scale bar: 100 µm. (D) Quantification of average cell diameter in pronephric tubules following the indicated treatments (**P<0.01, ***P<0.001). Data are mean±s.e.m.

Next, we addressed the molecular requirements for this process. Programmed cellular senescence has been linked to ERK signalling in the developing limb (Storer et al., 2013) and to TGFβ signalling in the context of the degenerating mesonephros (Muñoz-Espín et al., 2013). To test whether either of these signalling pathways is important for the induction of senescence in the axolotl pronephros, we treated axolotls with U0126, a specific inhibitor of ERK1/2 phosphorylation that is effective in salamanders (Yun et al., 2014), and LY2152799 (TGFβ inhibitor 1), a TGFβ receptor 1 kinase inhibitor (Muñoz-Espín et al., 2013), by daily compound administration to the water. In addition, we evaluated the effect of an alternative TGFβ inhibitor, SB-431542 (TGFβ inhibitor 2), which has previously been shown to inhibit TGFβ signalling in axolotls (Lévesque et al., 2007). Although senescent cells within the axolotl pronephros exhibit high levels of phopshorylated ERK (Villiard et al., 2016), we found that inhibition of ERK activation had no effect on the appearance and intensity of SAβgal activity in the developing pronephros (Fig. 2A,B), suggesting that induction of programmed senescence does not require ERK activation. By contrast, TGFβ inhibition with either inhibitor 1 or 2 led to a significant reduction in such activity compared with control treatments, as seen at various developmental stages (Fig. 2A-C). This was accompanied by an increase in structural integrity of the pronephric tubules of animals treated with the TGFβ inhibitors (Fig. S5). In addition, TGFβ inhibition resulted in a decreased average cell diameter within pronephric tubules (Fig. 2D), consistent with a reduction in cell senescence within these structures. Importantly, TGFβ inhibition also led to a delay in the onset of pronephros degeneration, which took place at stage 56 in animals treated with TGFβ inhibitor 1 (58.3%, *n*=12), as opposed to stage 54 in all of the control treated animals (*n*=10). We conclude that programmed cellular senescence in the developing axolotl pronephros is independent of ERK/MAPK activation, but is dependent on TGFβ signalling. Together, these observations suggest that, as in mammals, cellular senescence occurs in a programmed manner during axolotl development and contributes to the degeneration of the functional embryonic kidney, a key physiological process during embryogenesis.

### Cellular senescence in *Xenopus* development

**Fig. 3.**
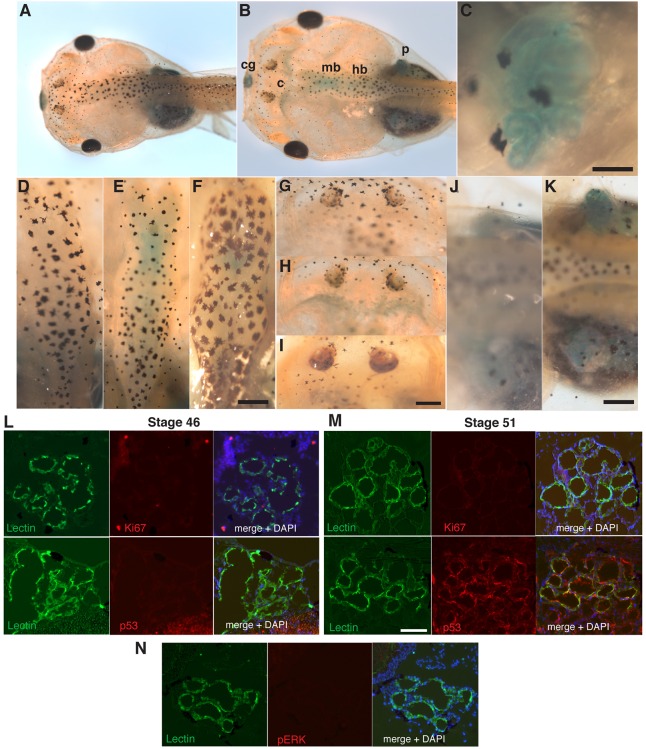
**Programmed cellular senescence during *Xenopus laevis* development.** (A-K) Representative images of SAβgal staining of whole-mount *Xenopus* embryos at the indicated developmental stages (*n*=8 per stage). (A,B) *Xenopus* embryos, NF 46 (A) and NF 49 (B). (C) *Xenopus* pronephros, NF 49. (D-F) *Xenopus* brains at NF 46 (D), NF 49 (E) and NF 52 (F). (G-I) *Xenopus* anterior cartilage at NF 46 (G), NF 49 (H) and NF 52 (I). (J,K) *Xenopus* pronephros at NF 46 (J) and NF 49 (K). Senescence is absent in brain and cartilage at NF 46 and NF 52. c, cartilage; cg, cement gland; hb, hindbrain; mb, midbrain; p, pronephros. Scale bars: 100 µm in C; 50 µm in D-K. (L-N) Representative cryosections of *Xenopus* pronephros at NF 46 (SAβgal negative, L) and NF 51 (SAβgal positive, M and N) following staining with *E. cristagalli* lectin, DAPI and the indicated antibodies. Scale bar: 50 µm.

**Fig. 4.**
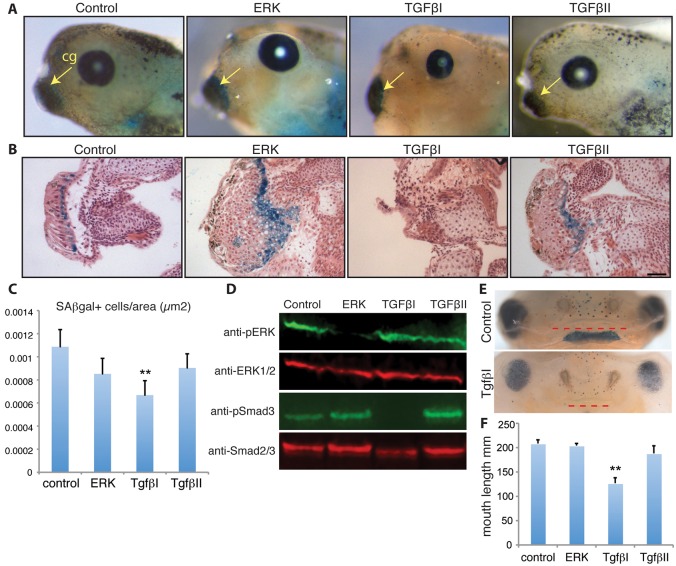
**Inhibition of TGFβ signalling abrogates cellular senescence in *Xenopus laevis* cement gland, leading to morphogenetic alterations in neighbouring structures.** (A) SAβgal staining of whole-mount *Xenopus* embryos at NF 45 following treatment with the indicated inhibitors, from stage 24 onwards. Arrows indicate the cement gland. Positive signal is present in the control tadpoles (*n*=6). (B) Representative paraffin wax-embedded sections following SAβgal/Haematoxylin and Eosin co-staining. Senescent cells are absent and the cement gland is smaller in animals treated with the TGFβ inhibitor 1. Scale bar: 50 µm. (C) Quantification of number of SAβgal-positive cells per area (µm^2^) in NF 45 tadpoles following treatment with the indicated inhibitors, from stage 24 onwards (*n*=4, **P<0.01). Data are mean±s.e.m. (D) Western blot analysis of tadpole extracts following the indicated treatments. Phospho-Smad3 levels decrease only upon treatment with TGFβ inhibitor 1 (*n*=5). (E) Representative images of the anterior region of *Xenopus* embryos at NF 45 following the indicated treatments. Abrogation of cellular senescence through TGFβ inhibition results in a substantial shortening of the mouth (red dotted lines) (*n*=8). (F) Quantification of average mouth length in NF 46 tadpoles treated with vehicle (DMSO) or TGFβ inhibitor 1 (*n*=8, **P<0.01). Data are mean±s.e.m.

To address whether programmed cellular senescence is also part of the developmental programme in anuran amphibians, we analysed its occurrence during the development of *Xenopus laevis*. SAβgal staining of *Xenopus* tissues revealed distinct and reproducible areas of cellular senescence during defined developmental time windows (Fig. 3). These areas were evident at Nieuwkoop and Faber (NF) stage 49, and corresponded to specific structures, including the tissues surrounding the cement gland (Fig. 3B), the midbrain and hindbrain (Fig. 3B,E), anterior cartilage (Fig. 3B,H) and, as previously observed in the axolotl (Fig. 1), the pronephros (Fig. 3B,C,K). Except for the cement gland, which was positive at NF 45 (Fig. 4) and remained so until its disappearance at the end of NF 49, all structures were negatively stained at NF 46 and earlier developmental stages (Fig. 3D,G,J). In addition, SAβgal staining had almost disappeared from brain and cartilage structures by NF 52 (Fig. 3F,I), highlighting the transient nature of the senescent cells. A developmental window was also observed in the case of the pronephros, which became senescent between NF 46 and NF 49 (Fig. 3C,K and Fig. S6) and remained as such until its degeneration was completed at stage 58, consistent with our observations in the axolotl (Fig. 1, Fig. S1) and with previous observations in mammals (Muñoz-Espín et al., 2013) and birds (Nacher et al., 2006). Importantly, both the pronephros (Fig. 3L,M) and the cement gland (Fig. S7) exhibited other hallmarks of senescence that are concomitant with SAβgal activity. These included lack of proliferation markers, such as Ki67, and increased protein levels of p53, as determined by immunofluorescence in sections co-stained with the lectin *Erythrina cristagalli*, which binds strongly to various *Xenopus* structures, including the pronephros and cement gland. Together with our observations in the axolotl, these data suggest that cellular senescence is induced in a programmed manner during amphibian development.

To investigate the molecular determinants and functional relevance of cellular senescence during *Xenopus* development, we focused on the cement gland, a transient mucus-secreting organ that allows larvae to attach to a substrate before they can swim or feed (Sive and Bradley, 1996). Through SAβgal staining, we identified a one- to three-cell layer of senescent cells surrounding the columnar epithelium that constitutes the cement gland (Fig. 4A,B, Fig. S7). Notably, these cells exhibit high levels of activated ERK1/2 (Fig. S7), a known regulator of senescence. Furthermore, regulators of TGFβ signalling, which is required for cell senescence in the axolotl pronephros, are known to be expressed in the area surrounding the cement gland during its development (Kondaiah et al., 2000). To address the molecular requirements for the induction of senescence within these tissues, we exposed embryos to ERK or TGFβ inhibitors from NF 24 to NF 45. Exposure to TGFβ inhibitor 1 led to a significant reduction in the number of senescent cells in the cement gland area, whereas ERK inhibition or treatment with TGF inhibitor 2 had no effect (Fig. 4A,B,C). Of note, both the ERK and TGFβ inhibitor 1 were effective at inhibiting their targets in this experimental context, as demonstrated by western blot detection of phospho-ERK1/2 and phospho-SMAD3 forms (Fig. 4D). In addition, ERK inhibition led to a marked reduction in the levels of active, phospho-ERK1/2 specifically in the cement gland, as demonstrated through immunostaining (Fig. S7). Despite its efficacy in axolotls (Fig. 2B), TGFβ inhibitor 2 was not able to inhibit its target in *Xenopus* (Fig. 4D), suggesting that this inhibitor is not effective at limiting TGFβ signalling and providing an explanation for the lack of phenotype in the treated animals. These observations suggest that, as in the case of the axolotl pronephros, developmentally programmed senescence in the cement gland depends on TGFβ signalling.

Finally, we examined the phenotype of embryos deficient in senescent cells due to temporary TGFβ inhibition. We observed that such animals exhibited a smaller cement gland and defects in its neighbouring tissues, including elongation of the nostrils and a substantial reduction in the length of the primary mouth, when compared with control animals (Fig. 4D). The defects in oral cavity morphogenesis were specific, as neither ERK inhibition nor treatment with the TGFβ inhibitor 2 led to significant changes in mouth length (Fig. 4E). Furthermore, this phenotype was accompanied by feeding impairments, as reflected by a reduction or absence of algae intake during a 3 h feeding period. Thus, loss of senescent cells due to TGFβ inhibition results in marked defects in the development of the cement gland and adjacent anterior structures.

These data suggest that TGFβ is required for developmental senescence in the cement gland, and for morphogenesis of this and other crucial structures in the surrounding area. Although it is not yet possible to dissociate unequivocally the effect of abrogating senescence from overall TGFβ inhibition in this context, as was the case with similar experiments in mammals (Muñoz-Espín et al., 2013; Storer et al., 2013) and indeed in the axolotl pronephros, these results raise the possibility that TGFβ-dependent induction of cellular senescence in the cement gland contributes to the organogenesis of the cement gland itself and that of adjacent structures such as the oral cavity.

## DISCUSSION

Here, we show that cellular senescence is a key feature of the developmental programme in amphibians. Our results reveal that cell senescence occurs in specific embryonic structures during well-defined time windows. These include the pronephros, in the case of axolotls (order Urodela), and the cement gland, pronephros, anterior cartilage, midbrain and hindbrain in the case of *Xenopus* (order Anura). It is possible that senescence induction could occur during the development of additional structures, as illustrated by the findings of Villiard et al. (2016). In the case of the pronephros and the cement gland, we show that programmed senescence is required for the physiological degeneration of the former and for the appropriate development of the latter.

Whereas the occurrence of programmed senescence during the development of the embryonic kidney and regions of the brain is consistent with previous reports in mammals (Muñoz-Espín et al., 2013; Storer et al., 2013) and birds (Nacher et al., 2006; Storer et al., 2013), our findings also suggest that senescence occurs during the development of structures that are not present in amniotes, such as the cement gland. Furthermore, despite the high degree of conservation in the molecular mechanisms underlying limb development, we had previously found that cell senescence is not induced in the developing limb in salamanders (Yun et al., 2015), in contrast to observations in mice (Storer et al., 2013). In the developing mouse limb, senescent cells are found within the interdigital webs and have been proposed to contribute to their apoptosis-mediated degeneration during digit formation (Storer et al., 2013). Notably, interdigital cell death is a developmental mechanism only found in amniotes (Cameron and Fallon, 1977). In view of these differences, it is possible that cellular senescence has been incorporated into the developmental programme at various different times during vertebrate evolution. From a functional perspective, our work suggests two roles for cell senescence during amphibian development: tissue degeneration, as exemplified by the pronephros; and remodelling of larval structures, as exemplified by the cement gland.

In the case of the pronephros, we find that cellular senescence is induced in a TGFβ-dependent manner, spreads throughout the structure over time and leads to the eventual degeneration of the structure through an apoptotic and macrophage-dependent mechanism. This overall process shares many similarities with the degeneration of the mammalian mesonephros (Muñoz-Espín et al., 2013). Notably, the induction of cell senescence during pronephros degeneration, the directionality of its spreading and its dependence on TGFβ are key shared features. Nevertheless, we also found differences in the degeneration process between the mammalian mesonephros and the amphibian pronephros. Most notably, degeneration of the axolotl pronephros takes place over a much longer period (over 8 weeks) and is accompanied by apoptosis, concomitant with recruitment of macrophages at stage 54. Thus, it resembles the situation in the apical ectodermal ridge of the mouse limb, in which the ultimate fate of senescent cells is apoptosis and macrophage-mediated clearance (Storer et al., 2013), rather than that of the mesonephros, where apoptosis does not take place (Muñoz-Espín et al., 2013). Furthermore, even though the peak of apoptosis is seen at stage 54, the axolotl pronephros does not fully disappear until late stage 57, suggesting that this mechanism is not efficient enough to promote the clearance of the structure in its entirety. These findings are intriguing in the light of recent observations demonstrating that salamanders possess a highly efficient mechanism of macrophage-dependent senescent cell clearance that operates throughout lifespan and prevents accumulation of senescent cells (Yun et al., 2015). A possible explanation is that these studies focused on post-reproductive forms, which possess a fully developed immune system, and not on developing animals. Indeed, we speculate that these differences may be explained by the degree of maturation of the immune system at the point of degeneration, which could affect the abundance, activation and functions of the immune components involved in the process. In agreement with this idea, larval macrophages are morphologically distinct, do not express MHC class I molecules and respond differently to bacterial stimulation (Horton et al., 2003; Marr et al., 2005; Robert and Ohta, 2009). In addition, it is worth noting that natural killer (NK) cells are absent from developing amphibians until the onset of metamorphosis, at stage 57-58 (Horton et al., 2003; Robert and Ohta, 2009). Notably, this coincides with the time at which the pronephros disappears. As NK cells are known to mediate senescent cell clearance in various *in vivo* contexts (Xue et al., 2007; Krizhanovsky et al., 2008; Sagiv and Krizhanovsky, 2013), they could also be required for the elimination of senescent cells during the final stages of pronephros degeneration in developing amphibians.

In the case of the cement gland, our studies reveal that cellular senescence is induced in the immediate cell layers surrounding this structure in a programmed manner and suggest that this contributes to its organogenesis as well as to the remodelling of adjacent structures such as the oral cavity. The mechanisms by which cellular senescence functions in this context are still unclear, and it remains possible that senescent signals are required for organogenetic processes such as tissue movement, growth and patterning. Nonetheless, it is noteworthy that, as in the mesonephros (Muñoz-Espín et al., 2013), the induction of developmental senescence in the cement gland is dependent on TGFβ. This is consistent with the expression pattern of TGFβI in the immediate tissues surrounding the cement gland during its development (Kondaiah et al., 2000). TGFβ is a mediator of senescence both *in vitro* and *in vivo* (Kuilman and Peeper, 2009; Acosta et al., 2013; Muñoz-Espín et al., 2013). Notably, it is also required for the maintenance of the SASP, and as such mediates non-cell autonomous functions of senescent cells, such as inflammation, modification of the tissue microenvironment and paracrine senescence (Hubackova et al., 2012; Acosta et al., 2013). Thus, in the cement gland, it is possible that the TGFβ-dependent secretory phenotype mediates senescent cell effects on the cement gland itself, as well as on the adjacent oral cavity. Further efforts should focus on identifying the SASP components involved. As the opening of the primary mouth in *Xenopus* and mammals is controlled by Hedgehog signalling (Tabler et al., 2014), it is possible that SASP molecules modulate members of this pathway. In the pronephros, TGFβ could mediate the propagation of cellular senescence through a bystander effect, which has been shown to occur in salamanders (Yun et al., 2015). Yet, owing to the lack of effective ways of selectively targeting senescent cells, for all senescence mediators analysed here and elsewhere it is difficult to separate the effect of senescence elimination from that of the mediator itself. This should be addressed by devising strategies for specific senescent cell targeting.

It is noteworthy that in both *Xenopus* and axolotl structures, senescent cells exhibit upregulation of p53 levels, raising the possibility that p53 could play a role in developmental senescence. This is interesting in the light of reported findings in mammals indicating that developmental senescence is p21 dependent but p53 independent (Muñoz-Espín et al., 2013; Storer et al., 2013). Although p53 is non-essential during mammalian development, a result often attributed to the existence of functional redundancy between p53 family members, it plays crucial roles during the development of amphibians, in which p63 and p73 are not expressed (Wallingford et al., 1997; Yun et al., 2013). Thus, it is possible that p53 and its family members could have functions in developmental senescence, and that these would be more apparent in the amphibian context thanks to its lack of redundancy. Alternatively, a dependence on p53 in the amphibian case could constitute an evolutionary divergence. Further research should explore the role of p53 and its family members as regulators of senescence in development.

To the best of our knowledge, our findings, together with those of Villiard et al. (2016), constitute the first demonstration of cellular senescence in amphibian development. They reinforce the concept that cellular senescence is a process intrinsic to the developmental programme, and provide new functional insights into its specific roles within it. Moreover, they extend the participation of cellular senescence in organogenesis from amniotes (such as mammals and birds) to all tetrapods, which is further extended to fishes (Villiard et al., 2016). In light of its conservation, we propose that programmed cellular senescence could constitute a developmental feature of all vertebrates. As such, it is possible that the functions of cell senescence in development arose early during evolution and could be central to the origin of other forms of cellular senescence.

## MATERIALS AND METHODS

### Animal husbandry and procedures

Procedures for care and manipulation of all animals used in this study were performed in compliance with the Animals (Scientific Procedures) Act 1986, approved by the United Kingdom Home Office. Axolotl eggs (*Ambystoma mexicanum*) were obtained from Neil Hardy Aquatica (Croydon, UK) and maintained in aquaria at 18-22°C with a 12 h light/dark cycle. Axolotl larvae were anesthetised in 0.02% tricaine prior to end point. Axolotl developmental stages were defined as described previously (Schreckenberg and Jacobson, 1975; Nye et al., 2003). *Xenopus laevis* embryos [kind gifts from Roberto Mayor (University College London, UK) and Jeremy Green (King's College, London, UK)] were maintained in normal amphibian medium [NAM: 110 mM NaCl, 2 mM KCl, 1 mM Ca(CO_3_)_2_, 1 mM MgSO_4_, 0.1 mM EDTA, 1 mM NaHCO_3_, and 2 mM sodium phosphate] 1:10 changed daily, in aquaria at 18-22°C with a 12 h light/dark cycle. From stage 46 onwards, larvae were fed a mixture of spirulina powder (ZM systems) and Tetramin baby (Tetra). Animals were anesthetised in 0.05% tricaine prior to end point. *Xenopus* developmental stages were defined as described by Nieuwkoop and Faber (1994).

### Inhibitor treatments

Axolotl or *Xenopus* embryos were kept under normal aquarium conditions until stages 30 and 24, respectively, and then treated with 50 µM UO126 (an ERK inhibitor; Merck), 50 µM SB-431542 (a TGFβR1 ALK5/4/7 inhibitor; BioVision), 50 µM LY2157299 (a TGFβR1 kinase inhibitor; Cayman) or DMSO as control treatment. All compounds were administered directly to the aquarium water or in a 1:10 NAM:H_2_O solution at the desired concentrations. Solutions were changed every day for the duration of the experiment. All treatments were performed in the dark and stopped at stage 46 (axolotl) or 45 (*Xenopus*).

### Senescence-associated β-galactosidase staining

For detection of SAβgal in animal tissues, axolotl or *Xenopus* embryos were fixed for 15 min in 0.25% glutaraldehyde, washed three times in PBS and stained overnight using the SA-β-gal kit (Cell Signalling) according to the manufacturer's instructions. Stained samples were then washed in PBS, fixed in 4% PFA and embedded in Tissue Tek-II (for cryosectioning) or in paraffin wax (for immunohistochemistry).

### Immunohistochemistry

Paraffin wax-embedded tissue sections (8 µm) were deparaffinised in xylene and rehydrated through a series of alcohol treatments. Endogenous peroxidase activity was neutralised by incubation in 3% hydrogen peroxide (Sigma-Aldrich) for 5 min. Sections were permeabilised in 0.2% Triton X-100/PBS, blocked in 10% goat serum and incubated with the indicated primary antibodies [anti-kidney 4A6.2C10, European Xenopus Resource Centre; anti-axolotl p53 (Yun et al., 2013) 1:200] overnight. In all cases, anti-mouse or anti-rabbit HRP-conjugated antibodies (DAKO) were used for secondary staining (1:1000, 1 h). Peroxidase activity was detected using the 3,3′ diaminobenzidine (DAB) substrate kit (BD Pharmingen). Samples were mounted in Vectashield mounting medium (Vector Labs) and observed under a Zeiss AxioZoom stereomicroscope (Zeiss). Image analysis was performed using Zen Pro Software (Zeiss). For each sample, 20-30 sections were analysed.

### Cryosectioning

Whole-mount stained samples were embedded in Tissue Tek-II and serially sectioned in a cryostat (Leica) at 12 µm. Sections were collected in Superfrost slides and stored at −30°C until use.

### Immunofluorescence

*Xenopus* tissues were fixed in 4% ice-cold paraformaldehyde (PFA) for 16-18 h at 4°C, washed twice in PBS and embedded in Tissue Tek-II for cryosectioning, as described above. Sections were hydrated in TBS, permeabilised in 0.05% TritonX-100/PBS for 10 min and incubated in 50 µg/ml fluorescein-conjugated *Erythrina cristagalli* lectin solution (Vector labs). Antibody staining of tissue sections was performed as described previously (Yun et al., 2013). Sections were incubated with the indicated primary antibodies overnight: anti-Ki67 (Cell Signalling, 1:100), anti-phospho-ERK Thr202/Tyr204 (Cell Signalling 4370, 1:200) and anti-p53 (1:200). In all cases, anti-mouse or anti-rabbit AlexaFluor488 and AlexFluor594 antibodies (Invitrogen) were used for secondary staining. Hoechst 33258 (2 µg/ml) was used for nuclei counterstaining. Samples were observed under a Zeiss Axiskop2 microscope and images were acquired with a Hamamatsu Orca camera using Openlab (Improvision) software.

### Cytochemistry

Granulocytes (including neutrophils) were detected by specific staining of tissue cryosections with the naphtol AS-D chloroacetate esterase kit (Sigma-Aldrich), while monocytes/macrophages were identified using the α-naphtyl acetate esterase (NAE) kit (Sigma-Aldrich), as previously described (Yun et al., 2015).

### TUNEL assay

Detection of apoptotic cells was performed in SAβgal-stained tissue paraffin or cryosections using the ApopTag Peroxidase In Situ Apoptosis Detection Kit (Millipore) following the manufacturer's protocol with minor modifications. The sections were incubated for 4 h at room temperature with digoxigenin-dNTP/TdT enzyme mix. Sections were mounted in DAKO mounting media with DAPI.

### EdU incorporation assays

To determine the presence of proliferating cells within the axolotl pronephros, axolotl larvae were injected (i.p.) with 5 µl of 10 mM 5-ethynyl-2′-deoxyuridine (EdU). After 12 h, larvae were fixed in glutaraldehyde, stained to detect SAβgal as described and cryosectioned. Sections were post-fixed in 4% PFA for 10 min and stained using Click-iT Edu Alexa Fluor 594 Imaging kit (Life Technologies) according to the manufacturer's instructions.

### Immunoblotting

Protein extracts were prepared by mechanical homogenisation of cells or embryos in 0.02 M HEPES (pH 7.9), 0.2 mM EDTA, 1.5 mM MgCl2, 0.42 M NaCl, 25% glycerol, incubating for 30 min at 4°C and clearing the debris by centrifugation. The resulting extracts were analysed by SDS polyacrylamide gel electrophoresis and transferred to a nitrocellulose membrane (Whatman), which was incubated for 15 min in Odyssey blocking buffer (Li-cor Biosciences) and incubated with primary antibodies: rabbit anti-phospho-ERK1/2 (Sigma-Aldrich M9692), 1:1000; mouse anti-ERK1/2 (Cell Signalling 4695S), 1:200; mouse anti-Smad2/3 (Cell Signalling 3103), 1:500; rabbit anti-phospho-Smad3 (S423/425) (Abcam ab51451), 1:500; mouse monoclonal anti-cMyc (Sigma M4439), 1:5000; rabbit anti-p53 (Yun et al., 2013), 1:1000 overnight. The membrane was then washed twice in TBS, incubated with IR-labelled secondary antibodies, IRDye680 and IRDye800 (Li-cor biosciences, 1:10,000), against the corresponding species (Li-cor) and analysed using an Odyssey scanner (Li-cor biosciences).

### Statistical analysis

Animals were randomly allocated to each sample group. Sample size for each experimental group/condition (n) was determined using G*Power 3.1 and is indicated in each figure legend. Larvae exhibiting signs of compromised health, such as tail bending (rare in axolotls; occasionally found in *Xenopus* depending on the quality of the batch) were excluded from the analysis. Statistical analyses were performed using Prism 4.0, GraphPad Software (www.graphpad.com) and unpaired two-tailed *t*-tests were applied unless otherwise stated. All experiments were replicated in at least three independent occasions.
